# QM/MM Molecular Dynamics Studies of Metal Binding Proteins

**DOI:** 10.3390/biom4030616

**Published:** 2014-07-08

**Authors:** Pietro Vidossich, Alessandra Magistrato

**Affiliations:** 1 Department of Chemistry, Autonomous University of Barcelona, 08193 Cerdanyola del Vallés, Spain; E-Mail: vido@klingon.uab.es; 2 German Research School for Simulation Sciences, D-52425 Jülich, Germany; 3 CNR-IOM-Democritos National Simulation Center c/o, International School for Advanced Studies (SISSA/ISAS), via Bonomea 265, 34165 Trieste, Italy

**Keywords:** Car–Parrinello molecular dynamics, QM/MM simulations, enzymatic catalysis, peroxidases, ribozymes, beta-lactamases, alpha-synuclein, transition metals

## Abstract

Mixed quantum-classical (quantum mechanical/molecular mechanical (QM/MM)) simulations have strongly contributed to providing insights into the understanding of several structural and mechanistic aspects of biological molecules. They played a particularly important role in metal binding proteins, where the electronic effects of transition metals have to be explicitly taken into account for the correct representation of the underlying biochemical process. In this review, after a brief description of the basic concepts of the QM/MM method, we provide an overview of its capabilities using selected examples taken from our work. Specifically, we will focus on heme peroxidases, metallo-β-lactamases, α-synuclein and ligase ribozymes to show how this approach is capable of describing the catalytic and/or structural role played by transition (Fe, Zn or Cu) and main group (Mg) metals. Applications will reveal how metal ions influence the formation and reduction of high redox intermediates in catalytic cycles and enhance drug metabolism, amyloidogenic aggregate formation and nucleic acid synthesis. In turn, it will become manifest that the protein frame directs and modulates the properties and reactivity of the metal ions.

## 1. Introduction

The trace amounts of metals present in living organisms are actually essential for their good functioning. Indeed, it is estimated that half of the proteome of living organisms encodes for metal binding proteins [1]. Bioinorganic chemistry and related disciplines dedicate research efforts to understand the activity of these metal-containing proteins (from the atomistic to the physiological scale), specifying the role of the metal(s). In the last few decades, improvements in the determination of the high-resolution structures of proteins and other biomolecules and the development of spectroscopic tools for their characterization have considerably advanced our capabilities to investigate these systems. At the same time, molecular modeling, thanks to algorithmic and computational improvements, has become a valuable tool to access the structural and mechanistic details of metal binding proteins, which are out of reach by experimental means. Indeed, molecular modeling may access resolution (atomic) and time scales (fs) that are often not accessible experimentally.

Here, drawing from our own research work, we will demonstrate the capabilities of modeling, specifically of the so-called quantum mechanical/molecular mechanical (QM/MM) approach. Originally proposed in 1976 by Warshel and Levitt [2], the method has been further developed by others [3,4], and many popular quantum chemical codes now offer this capability. This methodology has become nowadays widespread, showing its ability to describe and predict chemical processes in complex environments. For this reason, it gained the Nobel prize in Chemistry in 2013 [5]. Details of the method will be given in the next section.

Mechanistic studies of metal-containing enzymes will certainly contribute to advancing our understanding of the mechanisms of life. For instance, genome replication and maintenance are key biological processes for species propagation, and the magnesium-dependent polymerase enzymes and ribozymes are involved in these processes [6,7]. Besides this fundamental interest, mechanistic studies are beneficial also to the areas of biomedicine and biocatalysis. From the medical point of view, three aspects should be considered. The first concerns the relation between metal ion concentration and diseases. This is, for instance, the case of neurodegenerative diseases, such as Alzheimer’s and Parkinson’s, which have been related to the malfunctioning of metal homeostasis, with Cu and Zn being particularly involved [8]. Secondly, metalloenzymes may be involved in mechanisms of drug clearance or drug resistance [9]. Examples include the family of heme cytochromes P450, particularly involved in the metabolism of drugs, and the Zn-containing enzymes β-lactamases, which impart resistance to β-lactams antibiotics [10]. Thirdly, organometallic compounds may be designed to bind biomolecules. For instance, one of the most used anticancer drug is a Pt-containing molecule (cisplatin), and the interaction of this molecule with DNA is responsible for its activity [11,12,13]. Several other metal-based drugs have been discovered, which interact with DNA or key proteins [14,15,16].

On the side of biocatalysis, the development of catalytic systems based on earth abundant elements, which may favor organic transformation selectively and under mild conditions, is a major objective in the chemical industry [17,18,19,20]. Enzymes, and metalloenzymes in particular, display such characteristics and are thus attractive targets for engineering. Certainly, heme enzymes constitute a well-known example of such attempts [21,22,23]. The selected applications presented in this review aim at providing an account of the potentialities and limitations of QM/MM approaches in some of the above-mentioned research fields. Further applications of this methodology to investigate biological systems have been reported in recent reviews [24,25,26,27].

## 2. QM/MM Calculations: An Introduction

The hybrid QM/MM method is a multiscale technique [2,3,4,28], which responds to two necessary requirements for the modeling of metal binding enzymes. The first, common to the modeling of any enzyme, is that the complexity of the system has to be taken explicitly into account. In fact, the protein frame poses geometrical constraints on the first metal coordination sphere, while the second coordination shell may contribute to the activity of the enzyme by properly orienting the substrate or facilitating the transfer of functional groups. Additionally, at a longer range, electrostatic effects may stabilize certain metal oxidation states or reaction intermediates and, thus, have a remarkable impact on the reaction mechanism. Realistic model systems, including the enzyme, substrates, solvent and counterions, are capable of taking all of these features into account and are therefore to be preferred with respect to simplified models, which mimic only the reactive centers. Such systems may be handled efficiently by molecular mechanics (MM). The energy expression of an MM force field consists of the sum of energy terms, which account for bonded (bonds, angles and torsions), as well as non-bonded (electrostatic and van der Waals) interactions (see Equation (1) for the AMBER force field [29]), and it is based on a series of predefined empirical parameters:

*E* = *E_bond_* + *E_el_* + *E_VdW_* (1)

Unfortunately, most MM force fields, not taking into account explicitly the electronic degrees of freedom, experience large difficulties in describing the metal environment accurately, although notable improvements have been achieved in this respect in the last few years [30,31,32,33,34,35,36,37]. In fact, the metal moiety subtly depends on the electronic structure of the metal, which is difficult to capture at the force field level. In addition, force fields do not account for bond breaking and forming events, which take place during catalysis. To address these issues, is it necessary to move to the parameter-free quantum mechanical (QM) approaches. Computational quantum chemistry tools are routinely used to investigate organometallic systems [38,39,40]. Among these, density functional theory (DFT) [41,42], because of its favorable scaling with the number of atoms and its reasonable accuracy, is the recommended method to tackle metal containing molecules [43,44]. DFT allows treating at a reasonable level of accuracy the correlation effects. In particular, static correlation effects are particularly important for the correct description of the transition metal moiety, and they are extremely computationally costly to treat with post-Hartree–Fock methods. Thus, extensive research has been dedicated to test and improve the performance of DFT when dealing with transition metal systems, and dedicated reviews cover the developments achieved in this field [43].

According to the Kohn–Sham formulation [42], the electron density *n*(***r***) is expressed in terms of the occupied orbitals *φ_i_*(***r***):

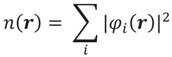
(2)
and the energy is given by:

*E* = min_{*φi*} _*E_KS _*[{*φ_i_*(***r***)}**;**{***R_I_***}] + *V_NN _*({***R_I_***}) (3)

where the E_KS_ is the sum of kinetic, nuclear-electron interaction, electron-electron interaction energy terms, while V_NN_ refers to the nuclear-nuclear interaction energy. The electron-electron interaction energy is divided into two parts: the classical Coulomb interaction energy between electrons and the non-classical part of the electron-electron interaction. The latter is included in the so-called exchange-correlation functional, E_xc_, of which only approximate forms are known. Because of its approximate nature, E_xc_ is the main source of error in DFT calculations, and the development of more accurate functionals has been an active area of research in the last few decades [43,45]. Some of the well-known deficiencies of the available E_xc_ include the neglect of dispersion interactions, which is responsible for the underestimation of interaction energies between, e.g., aliphatic or aromatic fragments, and the so-called self-interaction error, which is responsible for the unphysical electron delocalization experienced in some open shell systems [46]. Newly parameterized E_xc_ functionals [47], or the inclusion of empirical correction terms [48,49], are capable of better describing van der Waals complexes [43]. The inclusion of a fraction of exact (Hartree–Fock) exchange in the functional decreases the self-exchange interaction, improving the description of reaction barriers and radicalic systems [43]. However, in problematic cases, specific corrections have to be introduced as a remedy to the self-interaction error [50,51]. DFT is a ground state theory, but extensions to treat excited states are available. Among these, time-dependent DFT (TD-DFT) found widespread application for the study of electronic transitions [52,53].

Unfortunately, despite efforts to develop more efficient schemes (most notably, linear scaling methods) [54], the treatment of very large systems, such as a whole protein, by QM methods is not yet possible, although some notable examples have been reported [55]. Thus came the idea from Warshel and Levitt [2] to couple the two approaches, the QM and the MM, in order to accurately model the chemistry at the enzyme active site (QM subsystem), while keeping the environmental effects described at the MM level. Since the original proposal, researchers have proposed different schemes to couple the QM and the MM subsystems (see, e.g., [28] for a comprehensive account).

The examples presented here were based either on the Hamiltonian coupling scheme developed by Roethlisberger and coworkers [56,57] or on the multi-grid approach proposed by Laino *et al.* [58,59].

In both cases, the energy of the system is expressed as:

*E* = *E_QM_* + *E_MM_* + *E_QM/MM_* (4)

where E_QM_ and E_MM_ are the energies of the QM and MM subsystems, respectively, and E_QM/MM_ is the coupling between the two. The electrostatic contribution to E_QM/MM_ is:

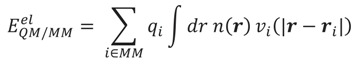
(5)
where *v_i_(**r**)* is a modified Coulombic potential, which prevents unphysical electron localization on the MM point charges [57,58] (the so-called spill-out effect). In order to reduce the computational cost of evaluating Equation (5), Rothlisberger and coworkers developed a multilayer scheme, in which Equation (5) is computed for the MM atoms closer to the QM region and a multipolar expansion is used to couple the QM region to more distant MM atoms [56,57,60]. Laino *et al.* developed, instead, a real space multigrid approach, in which the electrostatic potential is decomposed in terms of Gaussian functions with different cutoffs, and these contributions mapped onto grids of different spacings [58,59]. In both cases, van der Waals interactions between QM and MM regions are accounted for by the MM terms. The same holds true for bending and torsional terms across the QM/MM boundary. Particular attention has to be paid when the partition between the QM and the MM region cuts chemical bonds [28]. In such cases, the QM subsystem has to be properly saturated [61]. An often-adopted solution is to use a hydrogen link atom. This, however, introduces fictitious electrostatic interactions between the link atom and the MM region, and care has to be taken to avoid that these interactions affect the electronic structure of the QM region [60].

In mechanistic studies of chemical systems, the objective is to reconstruct the dependency of the energy on the nuclear coordinates ***R****_I_*. One approach to this problem is to scan the potential energy, *i.e.*, compute the energy for different atomic configurations. Efficient algorithms have been devised in order to locate the stationary points of the potential energy surface, which are the ones of most interest, as they correspond to the stable states and the transition state [62,63]. The applications presented in this review were instead based on molecular dynamics (MD) QM/MM simulations [64]. In this scheme, Newton’s equations of motion (Equation 6) are solved numerically [65].

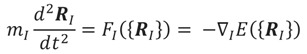
(6)
This approach is more appropriate for complex systems, and it allows accounting for finite temperature effects. When the forces are computed from a QM potential (here, the DFT energy functional), the procedure is known as *ab initio* molecular dynamics (AIMD) [66]. Assuming the Born–Oppenheimer approximation valid, the forces may be computed after optimizing the wave function at each step during the dynamics (Born–Oppenheimer AIMD). To avoid this costly evaluation, Car and Parrinello developed an efficient and accurate scheme, according to which the orbitals are treated as classical particles and are propagated simultaneously with the ions [67].

In the field of molecular modeling of complex systems, we would like to describe biochemical processes with realistic model systems and to follow their evolution in time and at finite temperature. As outlined above, the QM/MM approach allows tackling the size problem, such that the system of interest can be investigated by taking fully into account environmental effects. Unfortunately, the time-scale accessible by QM/MM MD simulations is limited by the costly evaluation of forces from electronic structure calculations (the QM part of the QM/MM potential). It thus appears that rare phenomena, such as chemical reactions and conformational changes, are not accessible via direct AIMD simulations.

Fortunately, statistical mechanics techniques may conveniently be used to address this issue. Metadynamics is a flexible and efficient approach to enhance the sampling of conformational space [68,69]. By means of a history-dependent biasing potential, the system is encouraged to visit new states, and the (negative of the) biasing potential constitutes an estimate of the underlying free energy surface. This approach is particularly useful to find the most likely reaction path when the reactive process involves complex collective reorganizations, in which the order of events is unknown. Thermodynamic integration [70,71] and umbrella sampling [72,73] simulations, among many other computational approaches [28], are also suitable to recover the free energy profile when the reaction path is known.

Many popular quantum chemical codes now include the possibility of performing QM/MM calculations. Because of the need to propagate the equations of motion several thousands of times, highly efficient codes are required to perform QM/MM-based AIMD simulations. The authors are more familiar with the CPMD [74] and CP2K [75] program packages, which were designed for atomistic simulations of large systems, and the applications presented in this review are mostly based on these codes. Both codes are particularly suited to high-performance computing resources, but display good performances on general-purpose clusters provided with tightly-coupled interprocess communications. For a list of other codes that can be applied to biological systems, see [28,76,77].

## 3. QM/MM Applications to the Study of Enzymatic Reactivity and Metal Binding to Biomolecules

In the following, we will review some of our recent results from the QM/MM MD modeling of metal-containing biomolecules. We will first address iron chemistry in the hydroperoxidase family of enzymes. Then, the catalytic role of Zinc in β-lactamases will be reported. The last two examples will highlight the role of the copper and magnesium binding on the conformational properties of α-synuclein and the active site geometry of a ligase ribozyme, respectively.

### 3.1. High Redox Intermediates in Enzymatic Cycles: Heme Hydroperoxidase Catalysis

Iron is the most abundant transition metal in living organisms [78]. Most of it is bound to the oxygen transport protein, myoglobin, or is stored by ferritin [79]. A small percentage of iron is bound to enzymes. Iron-containing enzymes perform diverse reactions, including oxidation, oxygenation and electron transfer reactions, exploiting the redox properties of the metal [80,81,82]. The nature of the coordinating ligands (first coordination sphere) has a profound effect on the redox properties of Fe. Furthermore, as exemplified by heme enzymes, the active site environment (second coordination sphere) plays a key role in directing the reactivity of the cofactor [83]. Heme hydroperoxidases are oxidoreductases that feature a heme cofactor (specifically, heme b in the systems analyzed below) and require hydrogen peroxide (H_2_O_2_) [84]. The catalytic cycle of this family of enzymes involves the following species: the resting ferric Fe(III) state and the so-called Compound I (Cpd I) and Compound II (Cpd II). Cpd I is the catalytically competent species, which forms upon the reaction of the ferric enzyme with H_2_O_2_ (Equation (7)). Cpd I has been characterized as an oxoferryl porphyrin cation radical (Porph^*^-Fe^(IV)^ = O), although in some hydroperoxidases, a protein amino acid residue may take the cation radical character (this species is usually called Cpd I*; Equation (8)). In peroxidases, Cpd I is responsible for the one electron oxidation of organic substrates, being reduced to Cpd II, an oxoferryl species, by the first equivalent of the substrate (S; Equation (9)). The resting state is restored by the reaction of Cpd II with a second molecule of the substrate (Equation (10)). In catalases, Cpd I is used to oxidize a second molecule of H_2_O_2_ to form dioxygen (Equation (11)).

Por-Fe^(III)^ + H_2_O_2_ → Cpd I (Por^+^-Fe^(IV)^ = O) + H_2_O (7)

Cpd I (Por^+^-Fe^(IV)^ = O) + aa → Cpd I* (Por-Fe^(IV)^ = O) + aa^+^ (aa = protein amino acid) (8)

Cpd I (Por^+^-Fe^(IV)^ = O) + SH → Cpd II (Por-Fe^(IV)^ = O) + S^+^ + H^+ ^(S = substrate) (9)

Cpd II (Por-Fe^(IV)^ = O) + SH + H^+^ → Por-Fe^(III)^ + H_2_O + S^+^ (10)

Cpd I (Por^+^-Fe^(IV)^ = O) + H_2_O_2_ → Por-Fe^(III)^ + H_2_O + O_2_ (11)

In the following, we review the *ab initio* modeling of two key steps of the enzymatic cycle of hydroperoxidases: the formation of Cpd I in horseradish peroxidase (HRP) and the reduction of Cpd I in *Helicobacter pylori* catalase (HPC). Furthermore, we report on the characterization of Cpd I in catalase-peroxidase (KatG). Open shell iron porphyrins, with close laying spin states, may appear a severe test case for DFT-based modeling. Actually, it turns out that current DFT approaches perform with reasonable accuracy for the high redox intermediates investigated in these studies (Cpd I and Cpd II) and the hexa-coordinated Fe(III) species. Furthermore, also the peroxyl radical and molecular oxygen are properly described by standard DFT functionals, contrary to the case of the hydroxyl radical [51,85]. Fe(II) porphyrins, not covered in this review, and Fe(II) organometallic complexes, in general, are more problematic from the perspective of DFT, and in such cases, the adoption of the DFT + U correction may turn out to be beneficial without additional computational cost compared to standard DFT [86,87].

#### 3.1.1. Mechanism of Cpd I Formation in Peroxidases

Horseradish peroxidase (HRP) belongs to the family of plant peroxidases [84]. This is a highly studied enzyme on which much of our current understanding of the functioning of heme peroxidases is based (possibly together with cytochrome C peroxidase). HRP has applications in biochemistry for its ability to generate a detectable signal upon substrate oxidation [88].

**Figure 1:**
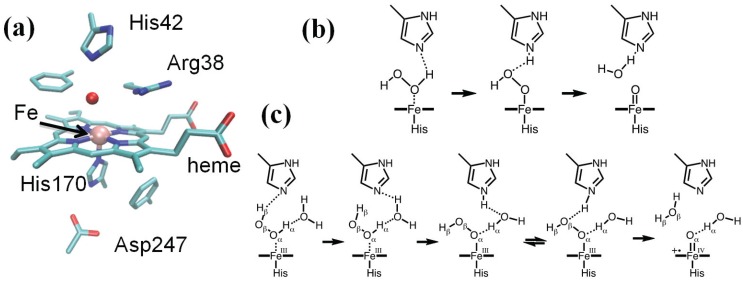
(**a**) HRP active site (PDB entry 1HCH); (**b**) the Poulos**–**Kraut mechanism; (**c**) the mechanism of Compound I (Cpd I) formation as reconstructed from a combination of quantum mechanical/molecular mechanical (QM/MM) calculations, Car**–**Parrinello and classical molecular dynamics.

The active site of HRP features the heme group axially coordinated to His170 (see Figure 1a), which, in turn, is hydrogen bonded to Asp247 [83]. On the heme distal side, His42 and Arg38 were shown by mutagenesis experiments to be involved in the formation of Cpd I, as variants lacking these residues display a decreased rate of Cpd I formation [89,90]. Poulos and Kraut determined the crystal structure of the related cytochrome C peroxidase and proposed a mechanism capable of rationalizing these findings (Figure 1b) [91]. According to them, the histidine residue on the distal side of the heme deprotonates H_2_O_2_, leading to the formation of a ferric hydroperoxide intermediate (Por-Fe^III^–OOH) that was later called Compound 0 (Cpd 0). Indeed, kinetic studies supported a reaction scheme in which at least one reversible intermediate is formed [92,93]. Based on the considerations of the acidities of the species involved, Jones and Dunford argued that the proton transfer from H_2_O_2_ to His42 should take place once the peroxide is bound to the iron [94].

The reaction was investigated by a combination of classical MD simulations, static QM/MM calculations and ab initio (QM/MM) MD simulations [95,96]. Such an integrated protocol allows addressing different aspects of the reactivity by the most appropriate and convenient approach. Thus, classical MD simulations based on the AMBER [29] force field were performed to investigate the conformational properties of the enzyme, including active site fluctuations and water accessibility. The model systems comprised the fully solvated enzyme with counterions, including about 66,000 atoms. QM (BP86 [97,98])/MM AIMD was used to investigate local fluctuations of intermediates for which force field parameters were not available. Finally, potential energy QM (B3LYP [99,100,101])/MM reaction scans were used to compute reaction paths and barriers for selected conformations from the MD simulations. The QM region included the porphyrin (excluding the propionate substituents), the iron proximal ligand, the distal His and Arg, the peroxide and a water molecule.

Compared to the Poulos–Kraut mechanism, our proposal (Figure 1c) features the assistance of one water molecule to facilitate proton transfer from H_2_O_2_ to His42 leading to Cpd 0, in which the His42(H^+^) hydrogen bonds with the catalytic water molecule. As revealed by ab initio Car–Parrinello MD simulations, His42(H^+^) easily exchanges the hydrogen bond partner to O_*β*_ of the peroxide, displacing the water molecule. Once His42–H^+^ interacts with O_*β*_ of the peroxide, it delivers the proton to it, leading to the concerted heterolytic breakage of the peroxide O_*α*_–O_*β*_ bond with the formation of Cpd I and a water molecule. This step displays the highest free energy barrier along the whole process (ΔF^#^ = 12.5–15 kcal/mol, depending on initial conformation). It is important to note that the direct proton abstraction by the distal His is characterized by a much higher barrier (ΔF^#^ = 20 kcal/mol) compared to the water-mediated process (5 kcal/mol), which was attributed to the long distance between His42 and the proximal proton of the Fe–H_2_O_2_ complex (N_*ε*_–H_*α*_ ≈ 4 Å). These findings highlight the role of classical molecular dynamics simulations to investigate protein fluctuations and the behavior of a solvent at the active site prior to the actual QM/MM modeling of the reaction. It was shown by classical MD studies that the active site of HRP is rather stiff, and His42 does not approach H_*α*_ closely [95], consistent with the (relatively low) B factors of the HRP distal residues in the native state and Cpd I [102]. Analysis of the dynamics of the water molecules in the heme pocket pointed to the occurrence of transient, though easily accessible, conformations more favorable for catalysis in which a water molecule comes to bridge H_*α*_ and His42 and facilitates the initial proton transfer.

#### 3.1.2. Characterization of Cpd I in Catalase-Peroxidases

**Figure 2:**
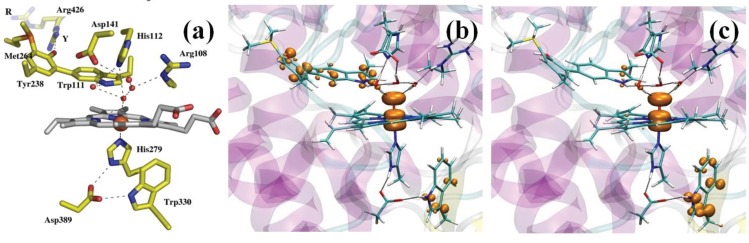
(**a**) KatG active site (PDB entry 1MWV); (**b**,**c**) the QM region is shown in stick representation together with an isosurface (orange surface) of the spin difference density distribution. (**b**) The catalytic Cpd I*: nearly one unpaired electron is found on the distal M-Y-W adduct when Y238 is deprotonated; (**c**) the peroxidatic Cpd I*: nearly one unpaired electron is found on the proximal W330 when Y238 is protonated.

Catalase-peroxidases (KatG) are bifunctional enzymes in which catalase activity is performed by a peroxidase-like active site [103]. As peroxidases show only little or no catalytic activity, rationalizing the activity of KatG remains an intriguing issue in peroxidase chemistry [104]. X-ray crystallography revealed a unique triad of covalently linked side chains of distal side residues, Trp111, Tyr238 and Met264 (*Burkholderia pseudomallei* KatG numbering throughout), the M-Y-W adduct (Figure 2a) [105], whose presence is required for catalase activity, as demonstrated by mutagenesis studies [103]. Tyr238, because of the extended π system and the possibility of associating with the mobile Arg426, displays a much lower pKa compared to normal tyrosines [106]. This observation prompted the investigation of the electronic structure of Cpd I as a function of pH [107]. Specifically, we considered the possibility of Tyr238 being protonated or not. Model systems comprised the protein fully solvated (about 137,000 atoms). The QM (BP86 [97,98]) region included the porphyrin (excluding the propionate substituents), the iron ligands, the side chains of residues His112, Arg108, the M-Y-W adduct and three water molecules forming H-bonds with distal side residues. The rest of the model was described by means of the AMBER force field.

Figure 2b,c shows the spin density distribution in Cpd I for the two protonation states of Tyr238. In both cases, a Cpd I* species results, in which Trp330 is in the radical state when Tyr238 is protonated, whereas the M-Y-W adduct has a radical character when Tyr238 is deprotonated. A Cpd I species as Cpd I* (Trp330^+^) is known to form in some monofunctional peroxidases, such as cytochrome C peroxidase and was observed by EPR spectroscopy in KatG [108,109]. On the contrary, a species as Cpd I* (Tyr238^+^) with an oxidation equivalent stored on the distal side of the heme was unprecedented. On the basis of these results, we were able to put forward a model of the enzymatic activity in which we postulated the formation of two Cpd I species, one of which is capable of peroxidatic activity, the other of catalytic activity. Importantly, the radical adduct species has been characterized spectroscopically very recently [110]. The peculiar properties of the M-Y-W adduct, specifically its low ionization potential when Tyr238 is unprotonated, have been proposed to be responsible for O_2_ activation by KatG [111].

#### 3.1.3. Mechanism of Cpd I Reduction in Catalases

**Figure 3:**
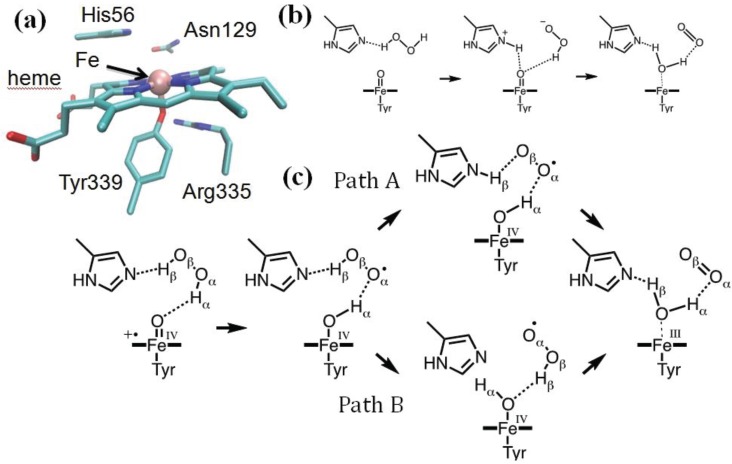
(**a**) HPC active site (PDB entry 2IQF); (**b**) the Fita–Rossmann mechanism; (**c**) the mechanism of Cpd I reduction as reconstructed from a Car–Parrinello/MM metadynamics simulations.

Heme catalases are used to decompose H_2_O_2_ to water and oxygen, thus protecting the organism from oxidative damage [112]. They are among the most efficient enzymes known, capable of degrading one million H_2_O_2_ molecules per second. *Helicobacter pylori* catalase (HPC) belongs to the family of small subunit catalases [113]. The active site of HPC features the heme group axially coordinated to Tyr339, which, in turn, is hydrogen bonded to Arg335 (Figure 3a) [114]. On the heme distal side, His56 and Asn129 are expected to participate in the catalytic reaction in which Cpd I is reduced back to the resting state by a second molecule of hydrogen peroxide, which provides the required two electrons and two protons (Equation (11)). Since the determination of the X-ray structure of a catalase, it was proposed that the reaction should proceed stepwise [115,116]. Specifically, Fita and Rossmann proposed that the distal His acts as an acid/base catalyst facilitating the transfer of a proton from the peroxide to the oxoferryl unit (Figure 3b). Because of this, an H^+^/H^−^ scheme was assumed, in which H^−^ is transferred directly to the oxoferryl unit and the transfer of H^+^ is mediated by the distal His.

Recent modeling of this catalytic step by means of QM/MM metadynamics simulations used a reduced model of the enzyme, including residues within 20 Å from the heme iron (about 4000 atoms). The QM (BP86 [97,98]) region included the iron-porphyrin with the methyl and vinyl substituents, the side chains of proximal ligands, Tyr339 and Arg335, the side chains of distal residues, Asn129, His56 and Ser95, the peroxide substrate and two water molecules. After exploring the reactant states for 2-ps simulation, about 6-ps metadynamics was used to probe the chemical steps and reconstruct the associated free energy profile. We showed that the reaction indeed proceeds via the Fita–Rossmann mechanism (Figure 3c) [85]. Nevertheless, the process consists of two one-electron transfers in the form of a hydrogen atom transfer and a concerted proton and electron transfer, and not by a proton and hydride transfer, as previously assumed. The first step consists of a facile hydrogen transfer from H_2_O_2_ to Cpd I, leading to Cpd II + HO_2_. The small energy cost for this hydrogen atom transfer is to be attributed to the short interatomic distances between donor and acceptor oxygen atoms [117], which can be attained in the active site of catalases thanks to the particular orientation of His56.

The conversion of Cpd II + HO_2_ to the resting state may take place via two competing pathways (Figure 3c). In one pathway (path A in Figure 3c), a proton is transferred from HO_2_ to His56, which then changes conformation, breaking the H-bond with the superoxide and forming a new one with the oxoferryl oxygen. This conformational change of His56 represents the highest energy-demanding step along this pathway (ΔF^#^ = 13 kcal/mol). The new conformation of His56 allows the facile transfer of the H_*β*_ proton, which occurs together with the passage of an electron from the superoxide to the oxoferryl. In the competing pathway (path B), a flip of the peroxyl radical reorients its proton towards the oxoferryl oxygen, facilitating a direct hydrogen atom transfer. Our analysis indicates that the basicity of His56 and the size of the distal site cavity are important factors governing the relative probability of the two pathways [85].

### 3.2. Zn Enzymatic Drug Metabolism: Antibiotic Hydrolysis by Metallo-β-Lactamases Enzymes

Zn is an essential metal ion in living organisms [118]. In bacteria, Zn ions contribute to one of the most important resistance mechanisms towards commonly used antibiotics [119]. Metallo-β-lactamases (MβLs) are broad-spectrum Zinc-dependent enzymes able to degrade most classes of β-lactam antibiotics (penicillins, cephalosporins and carbapenems) by catalytically cleaving their β-lactam moiety. MβLs are not sensitive to any of the available inhibitors, representing a serious clinical problem [10].

MβLs are divided into tree subclasses (B1, B2 and B3) and require one or two Zn(II) ions to be catalytically active. In the B1 and B3 subclasses, the active site has a potential tetrahedral Zn binding sites (ZnA) and a second tetrahedral/trigonal bipyramidal binding site (ZnB), which is common also to the B2 subclass. Despite the exact metal load necessary to cleave the antibiotics being unclear [120], the B1 and B3 classes seem to be active with one or two Zn ions [9,120]. The B2 subclass, instead, features a single ZnB metal ion and strongly prefers carbapenem antibiotics, which are key antibiotics against resistant Gram-negative bacteria.

QM/MM simulations have been used in the last decade to unravel the mechanism of several members of this family of enzymes [121,122,123,124,125,126,127,128,129]. Recently, Simona *et al.* [130], focusing on the enzymatic mechanism of CphA degradation from *Aeromonas hydrophila*, belonging to the B2 class, revealed interesting mechanistic features common to other classes of MβLs.

Starting from the crystallographic structure of CphA in complex with a partially hydrolyzed biapenem (Bia) [131] and, after having identified the most likely protonation state of ionizable residues [126], Simona *et al.* performed classical and hybrid QM (Car–Parrinello)/MM MD simulation studies of the complete hydrolysis reactions considering a different water content in the active site. The MM part (about 53,000 atoms) was treated with the parm99 AMBER force field [29], while the QM region, including His118 and His263 imidazole rings (cut at the Cγ), Asp120 and Cys221 (cut at the Cβ), the reactive part of the biapenem that is its backbone and part of its hydroxyethyl substituent at the 6 position, catalytic water (Wat-B) and, for ES2, the additional second water Wat2-A (59 and 61 atoms), was treated at the DFT-BLYP level [99,101].

**Figure 4:**
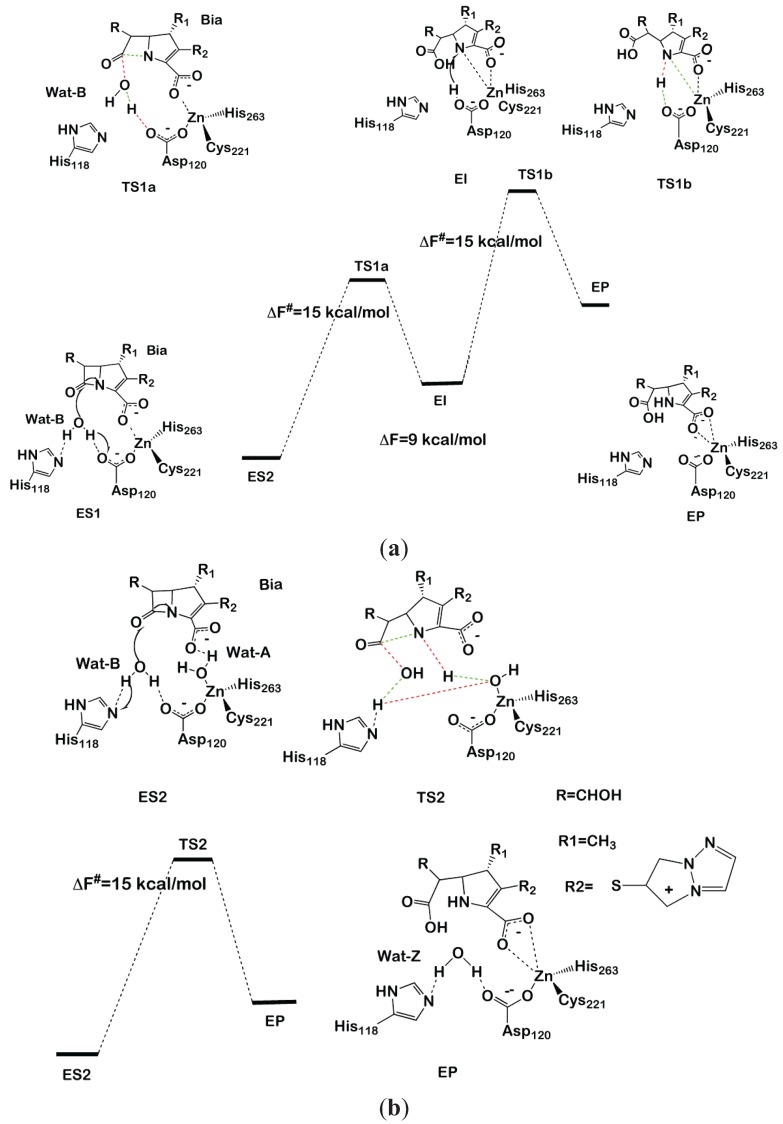
Reaction mechanism of biapenem hydrolysis proposed in [130]. In the transition states, bonds that are formed or broken are indicated as red and green dashed lines, respectively. The two-step and one-step reaction mechanisms are shown in (**a**) and (**b**), respectively.

In model ES1 (Figure 4a), the Zn is coordinated by Cys221, Asp120 and His263, with the carboxylate moiety of Bia completing the metal coordination sphere. In model ES2 (Figure 4b), instead, a water molecule (Wat-A) coordinates to Zn, replacing the carboxyl moiety. In both models, a water molecule (Wat-B) lies between His118 and Asp120, which are proposed to act as a H-bond acceptor of the nucleophile during the first reaction step.

After having equilibrated both models (ES1 and ES2) at the classical and, subsequently, at the QM/MM MD level, we proceeded to the modeling of the reaction by thermodynamic integration [70] using monodimensional reaction coordinates. For each of the three reaction steps considered (two for ES1 and one for ES2), 12 points were considered along the chosen reaction coordinate, each equilibrated for 3 ps of QM/MM MD. In ES1, we found that Asp120 activates the attacking water molecule by deprotonating it to form a hydroxide nucleophile. Subsequently, N of the β-lactam moiety coordinates to Zn, displacing the carboxylate fragment of Bia (Figure 4a). This step occurs with a free energy barrier (ΔF^#^) of 15 ± 3 kcal/mol. An intermediate forms, which lays 9 kcal/mol above the reactant state, featuring a distorted tetrahedral geometry in which Cys221, His263, N of the β-lactam and the Bia carboxyl group create the coordination sphere. In the second reaction step, Asp120 transfers the proton, abstracted from water in the first reaction step, to N of the β-lactam with ΔF^#^ = 15 ± 2 kcal·mol^−1^, resulting in an overall free energy barrier for the whole cycle of ΔF^# ^= 24 ± 3 kcal·mol^−1^, which is inconsistent with the experimental value (ΔG^#^ = 14 kcal/mol).

In contrast, the simulations performed on ES2 resulted in a complex set of chemical rearrangements at the transition state, which involves: (i) deprotonation of Wat-B by His118; (ii) Wat-A protonating N1, becoming transiently a Zn-bound hydroxide; and (iii) the transfer of a proton from His118 to the metal bound hydroxide, restoring Wat-A. This synchronous and complex exchange of protons leads in one step to the product state in which Wat-A replaces the position occupied by Wat-B at the resting state. The overall free energy barrier of this process is 15 ± 3 kcal/mol. This latter mechanism contrasts with previous computational studies, which point to a multistep process for the hydrolysis of biapenem [128]. Despite alternative reaction paths having been also suggested [128,132], it is of paramount importance to remark about the striking similarity between the reaction mechanism identified by Simona *et al.* and that catalyzed by B1 MβL CCrA in complex with cefotaxime [123]. In fact, despite CCrA being active as a di-Zinc form, in both cases, ZnB coordinates an auxiliary water molecule (here, Wat-A), which is not the active nucleophile, but anchors the β-lactam carboxyl group via H-bonding interactions, favoring an optimal orientation of the substrate in the active site. Moreover, upon nucleophilic attack of Wat-B, Wat-A protonates the nitrogen of the β-lactam moiety, allowing an efficient cleavage of the substrate in a single-step mechanism. In summary, modeling indicates that ZnB is a key player in β-lactam antibiotic hydrolysis [9,10] of different subclasses. This structural motif may be the target for drug design studies of inhibitors targeting simultaneously different MβLs subclasses. We believe that this, as well as other QM/MM studies, are examples of the ever-increasing role for hybrid quantum-classical approaches in drug design projects [133].

### 3.3. Cu-Mediated Amyloid Formation: Cu(II)-A-synuclein Adducts in Parkinson’s Disease

In the last decade, many research efforts have been devoted to elucidating the role played by metal ions in brain diseases. One of the reasons for this interest relies on the observation that metal ions, such as Fe, Cu and Zn, are involved in the onset of several neurodegenerative diseases, including Alzheimer’s, Parkinson’s’, amyloid lateral sclerosis and Prion’s diseases [134,135].

Despite their small amounts in the brain (0.5, 0.15 and 0.006 g per 1.5 kg, for Fe, Zn and Cu, respectively), these metals play essential functions, and their distribution is tightly regulated. Altered concentrations of metal ions result in toxicity [136]. Among the mechanisms proposed to rationalize the toxic effects induced by these metals is the accelerated formation of amyloids [136], which is *per se* at the basis of all neurodegenerative diseases [137]. According to this hypothesis, metal binding may induce conformational changes of the proteins related to the diseases, which, in turn, affect its aggregation propensity. Furthermore, Fe and Cu, being redox active, may produce reactive oxygen species, which may react and damage key proteins in the brain [138].

Among the possible neurodegenerative diseases enhanced by metal ions is Parkinson’s disease (PD). This is associated with the formation of α-synuclein (AS) aggregates in the Lewy body [139]. High Cu(II) concentrations have been observed in the cerebrospinal fluid of PD patients, suggesting that the disease may be associated with the presence of this metal, which is an AS aggregation enhancer [140]. Among the metal ions suspected to take part in the onset of PD, Cu is the most effective one in accelerating AS aggregation, being active already at µM concentrations [140].

In its monomeric forms, AS is a disordered protein, with no defined secondary structure, so that at physiological conditions, it can be described as an ensemble of structurally heterogeneous conformations [141,142].

A detailed understanding of the structural features, as well as of the coordination environment of Cu to AS is of paramount importance to elucidate the interactions at the molecular level between AS and Cu(II). However, the unstructured nature of AS makes it difficult to characterize these aspects from both the experimental and computational points of view [143].

Experimental EPR and NMR measurements investigating Cu(II) binding to AS suggested that the Cu(II) can bind in three regions of AS: (i) the N-terminal part with the highest affinity; (ii) His50; and (iii) the C-terminal (Asp119-Asp121-Asn122-Glu123, with lower affinity) [141,142]. In the highest affinity-binding site, the Cu is supposedly coordinated to Met1, Asp2 and a water molecule [144,145].

Here, we report on a computational study in which we employed classical and QM (Car–Parrinello)/MM MD simulations to investigate the binding of Cu(II) to AS [143]. This study constitutes an example of how the problem of determining the coordination environment and the structural features of metal binding to a disordered protein may be addressed by a combination of spectroscopic and computational techniques [143].

**Figure 5:**
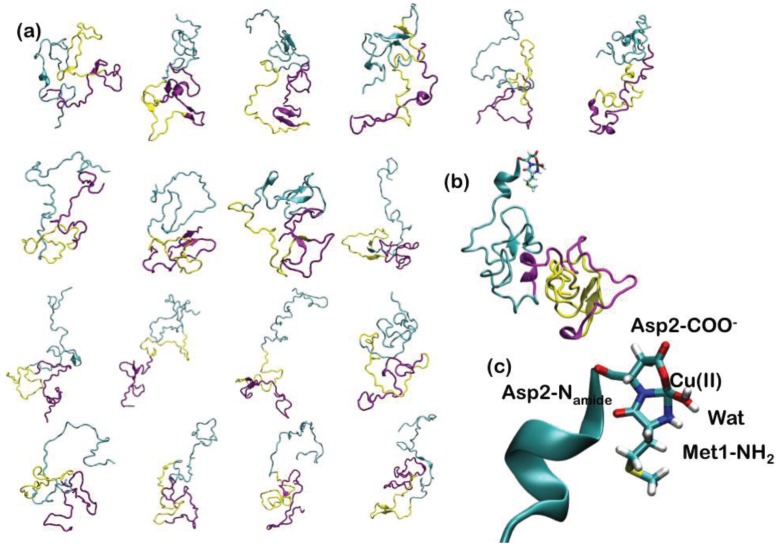
(**a**) The 18 most representative clusters extracted from the ensemble of conformations obtained from NMR experiments. α-Synuclein (AS) is a 140-amino acid protein divided into three regions: the N-terminal part, which comprises residues 1–60, the hydrophobic self-aggregation sequence (non-amyloid-beta component (NAC)) comprising residues 61–95 and the C terminal region. The N-terminal, NAC and C-terminal region are depicted in cyan, magenta and yellow ribbons, respectively; (**b**) Binding of Cu(II) to the N-terminal region of AS. (**c**) Close-up view of the coordination site.

Since AS is intrinsically unstructured, 18 representative NMR structures, covering about 50% of the conformational space spanned by the protein in solution, have been used to construct the adducts with Cu(II) (Figure 5). In the highest affinity binding site, Cu(II) has been imposed to bind to the N-terminal Met1 and Asp2 backbone nitrogen, Asp2 carboxylate side chain and a water molecule (Figure 5). The 18 Cu-AS adducts have been relaxed performing classical MD simulations (10 ns for each system), using the AMBER parm99 force field [29] and, subsequently, QM(Car–Parrinello)/MM MD (4 ps for each system). The QM part of the model consisted of Cu(II), the N-terminal Met-1 backbone unit and its side-chain up to the Cα atom, the entire Asp-2 residue and a water molecule coordinating the Cu(II) ion (27 atoms). This part was treated at the DFT-PBE [146] level, using a spin-unrestricted formalism. The average structural parameters, obtained as a weighted average over each representative conformers, depicted the metal binding site as a distorted tetragonal coordination geometry of Cu(II) (Table 1), in line with the literature structural data. Therefore, our data have provided an atomistic picture for the binding of Cu(II) to the putative highest affinity binding site of α-synuclein [143].

**Table 1:** Average values of bond lengths (Å) and angles (deg) for the Cu(II)-AS adducts calculated as the weighted average over the QM/MM MD trajectories of the 18 representative conformers. Standard deviations are given in parenthesis. @ refers to the residues to which the coordinating atom belongs. In the last column, the mean bond lengths and angles, obtained from the crystal structures of Cu(II)-peptide adducts, are reported [143].

Cu(II)-ligand bond lengths and angles for the AS-Cu(II)		X-ray
Cu(II)-NH_2_@Met1	2.06 (0.02)	2.00
Cu(II)-N^−^_amide_@Asp2	1.92 (0.02)	1.92
Cu(II)-O^−^@Asp2	2.01 (0.02)	1.98
Cu(II)-O@Wat	2.09 (0.02)	1.97
Asp2@O-Cu(II)-NH_2_@Met1	164 (2)	167
Wat@O-Cu(II)-N^−^_amide_@Asp2	168 (2)	166
N-amide-Cu(II)-NH_2_@Met1	84 (1)	84
Wat@O-Cu(II)-O^−^@Asp2	88 (1)	84

### 3.4. Mg(II) Ions in Nucleic Acids Synthesis: The Case of the RNA Ligase Ribozymes

Magnesium is the most abundant divalent cation in biological systems, and it is widely available in the biosphere (2% of the Earth’s crust) [147]. Mg(II) ions are involved in many aspects of cellular metabolism, including signaling, catalysis, structure stabilization and nucleic acids folding [147]. Among the functions played by magnesium in enzymatic reactions, we recall their impact on the binding of substrate to the active site of proteinaceous and ribonucleic acids enzymes, which may account for substrate binding specificity and for the modulation of chemical reactions [7].

Mg(II) ions are spectroscopically silent, and X-ray crystallography is the main experimental source of structural information on their location and coordination geometry. Unfortunately, experimental procedures during crystallization may favor/disfavor the occupancy of putative binging sites. Furthermore, in the crystallization procedure, the active metal ion is often replaced by catalytically inactive metals in order to trap the enzyme/substrate adducts [148]. Thus, the structural features of the active Michaelis complex may differ sensibly from those captured experimentally by metal ion replacement [148,149]. QM/MM MD simulations represent a reliable approach to investigate Mg(II) ion binding sites, capable of providing detailed structural information when only indirect experimental information on the residues forming the binding sites are available or to refine geometries obtained with chemical modifications of the real systems [6].

Recently, we provided a detailed structural characterization of the metal content and of the coordination sphere of the reactant and product states of a class I ligase ribozyme. RNA polymerase ribozymes are key actors in the RNA world hypothesis (according to this hypothesis, the critical event in the origin of life was the presence of an RNA molecule capable of self-replicating the RNA genome) [150] and for this reason attracted great research interest [151]. No naturally occurring polymerase ribozymes exist, and efforts to engineer them *in vitro* by evolution methods resulted in the creation of class I ligase ribozymes [151], and, later on, in a few examples of catalytically efficient RNA polymerases ribozymes [152,153]. Structural information on these RNA enzymes is limited, thus we focused on a class I ligase ribozyme, which has the same catalytic core of a polymerase ribozyme, but for which structural information is available [151,154].

**Figure 6:**
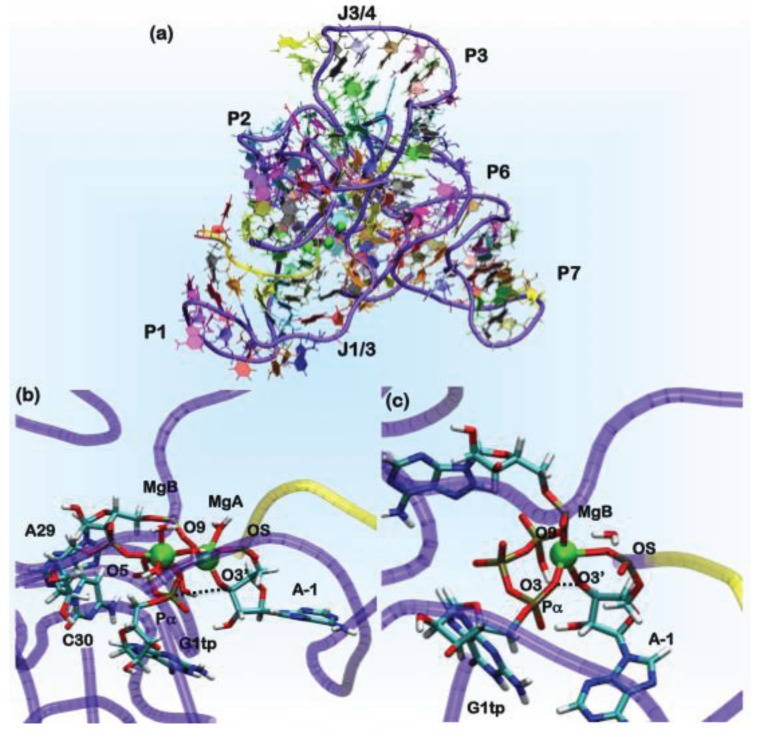
(**a**) The overall architecture of the ligase ribozyme, showing the relative domain orientations. In green spheres, the position of the Mg(II) ions is shown. In the yellow tube, the U-7-A-1 tract of the ribozyme is shown, which performs the nucleophilic attack on the P_*α*_ of the G1tp base. The rest of the ribozyme is shown as a violet tube. Nucleobases are colored by residue types. Representative structures of AdMgAB and AdMgB were obtained from QM/MM MD simulations, in (**b**) and (**c**), respectively. The ribozyme is represented in tube form following the same scheme mentioned above, while residues forming the binding site are shown in licorice and colored by the atom name.

In the crystal structure of the ligase ribozyme [151] (Figure 6), which represents the ligation product state, no metal ions were detected in the putative catalytic site. We applied different computational approaches with an increasing order of accuracy to provide a structural characterization of the Mg(II) ions in the catalytic pocket. We initially investigated three models of the product state using different Mg(II) concentrations [155]. The ions were initially placed in the most negative regions of the electrostatic potential, and classical MD simulations were performed using two representations for the Mg(II) ion in the catalytic site, namely the point charge model [156] (30 ns) and, later, the dummy cation approach, which allows for a more realistic description of the metal coordination geometry [157], (20 ns). The systems, comprising 63,000 atoms, were treated using the AMBER force field [29] along with the recent corrections for nucleic acids [158]. Among the three models considered, two displayed a Mg(II) located between A29, C30 and G1, the nucleobases indicated as forming the metal binding site of the metal by NAIM (nucleotide analogue interference mapping) experiments [155]. This site is referred to hereafter as MgA. Structural comparison between the different computational models bearing a Mg(II) in site A and the X-ray structure in terms of root mean square deviations (RMSD) and fluctuations (RMSF) allowed for checking the reliability of our computational protocol and choosing the most appropriate model for the ligation product state [155].

In the ligation product state, a single Mg(II) ion is present in the active site. This occurrence is consistent with what was observed in DNA and RNA polymerases. In these systems, believed to operate the polymerization/ligation reaction via a two-Mg(II) mechanism, the second ion should coordinate the pyrophosphate (PPi) moiety of the incoming nucleotide. In order to determine the catalytic competent form of the ribozyme, we have reconstructed the reactant state. This was done by cleaving the autoligation product in two parts: the U-7-A-1 fragment, representing the substrate, and the G1gtp-A121 part of the ribozyme (Figure 6). In G1, we reconstructed a guanosine triphosphate (G1tp).

In the search for potentially reactive adducts, we have considered different possible Mg(II) loads into the catalytic site. We first checked the stability of the adduct with only a single Mg(II) ion in the MgA site (model AdMgA). Surprisingly, in this configuration, the substrate does not stably bind inside the active site, as the distance between O3'@A-1, the nucleophile and P_*α*_, the atom undergoing the nucleophilic attack reaches a value of 6 Å already in the first hundreds of ps of classical MD simulations. Thus, we placed a second metal ion near the pyrophosphate moiety of G1tp at a distance of 4 Å from MgA. This second site is referred to in the following as MgB (model AdMgAB). Finally, we placed a single Mg(II) ion in MgB (model AdMgB). Both models resulted in being stable during the classical MD simulation. Thus, the modeling was extended to the QM (Born–Oppenheimer)/MM representation in order to account for the charge transfer and polarization effects. In these simulations, we treated the coordination spheres of the Mg^2+^ ions at the QM level. Sixty two atoms were included in the QM part for AdMgAB and 42 atoms for AdMgB. The QM part was treated at the DFT-BLYP level, and each adduct was simulated for 6 ps using the CP2K program [75]. The simulations of both the AdMgAB and AdMgB models provided O3'@A-1•••P_*α*_@G1tp distances, consistent with a reactive conformation of the adduct (Table 2) [155]. Comparisons of these coordination geometries of the catalytic site with the structures found in the MeRNA database and with those from previous computational studies were used to validate our results (Table 3) [159,160,161]. In fact, the coordination geometries of the modeled ligase ribozyme were in line with other polymerase enzymes or ribozymes, whose catalytic activity relies on a two-metal ion mechanism [162]. Since the AdMgA adduct turned out to be unstable, but the importance of A29 and C30 in the catalytic activity of this ribozyme has been observed experimentally, we suggest that a two-metal ion binding site is the most likely, consistent with experimental suggestions [151,154] and with the Steitz’s hypothesis [162].

**Table 2:** Average bond lengths (Å) of Mg(II) ions and the coordination ligands obtained from the QM/MM MD of the AdMgAB and AdMgB. Standard deviations are reported in parenthesis.

AdMgAB						AdMgB		
Atom-1	Atom-2	Distance (Å)	Atom-1	Atom-2	Distance (Å)	Atom-1	Atom-2	Distance (Å)
O^(R)^@C30	MgA	2.50 (0.06)	O3@G1_tp_	MgB	2.26 (0.11)	O3@ G1_tp_	MgB	2.12 (0.06)
O^(S)^@A29	MgA	2.06 (0.06)	O^(S)^@A29	MgB	2.26 (0.06)	O^(S)^@A29	MgB	1.86 (0.12)
O5@G1_tp_	MgA	2.06 (0.06)	O9@G1_tp_	MgB	2.98 (0.10)	O9@ G1_tp_	MgB	2.01 (0.06)
O@Wat2	MgA	2.35 (0.11)	O3’@A-1	MgB	2.00 (0.05)	O3'@A-1	MgB	2.46 (0.13)
O@Wat3	MgA	2.47 (0.07)	O^(S)^@A-1	MgB	2.17 (0.07)	O4@G1_tp_	MgB	2.26 (0.06)
O@Wat4	MgA	2.18 (0.10)	O@Wat1	MgB	2.28 (0.09)	O^(S)^@A-1	MgB	2.36 (0.05)
MgA	MgB	4.05 (0.0001)	O4@G1_tp_	MgB	2.67 (0.12)			
O3’@A-1		Pa@G1_tp_		4.08 (0.10)		O3’@A-1	Pa@G1_tp_	3.96 (0.20)

**Table 3:** Average bond lengths (Å) of Mg(II) ions and the coordination ligands obtained from the MeRNA database [161] and from other QM/MM studies [159,160].

X-Ray (MeRNA)		QM/MM MD
Mg-O3’	2.12 (0.17)	2.1–2.5
Mg-O@P	2.12 (0.17)	2.2–2.7
Mg-O@Wat	2.15 (0.18)	2.1–2.4

## 4. Conclusions

The QM/MM approach is particularly suited for the study of metal binding proteins and metalloenzymes, because it offers the possibility of describing accurately the intricate nature of the metal coordination sphere (by means of the quantum chemical description), yet maintaining a realistic description of the protein environment (treated at the computationally more accessible molecular mechanics level) [64].

The studies outlined in this review highlight the capabilities of the method in different aspects of biochemistry. Namely, we have reported mechanistic studies on the formation and reduction of high redox intermediates in heme enzymes and on the antibiotic degradation by Zn-dependent lactamases [130]. Furthermore, we have outlined how the QM/MM method, combined with classical MD simulations, allows one to refine the coordination environments and to test different metal loadings for the stability of the Michaelis complex of an RNA ligase ribozyme [155] or it may be used to investigate how metal coordination affects the conformational properties of peptides, as we showed for the copper-mediated amyloid formation of α-synuclein [143].

The reported examples highlight the interrelation between computational and experimental studies. The interplay between these two approaches is going to become even tighter in the near future. Indeed, thanks to advances in both software and hardware, the execution time of sophisticated QM/MM studies is being reduced, such that research projects integrating accurate virtual experiments may be designed.

The QM/MM approach addresses the problem related to the size of the biomolecular systems, by explicitly taking into account the whole system and describing it at different levels of accuracy, according to the relative importance of the different parts. However, the time-scale of this type of virtual experiment is still strongly limited by the computational intensiveness of QM calculations, and it should still be greatly expanded.

One approach to overcome this issue is to combine QM/MM modeling with classical molecular dynamics, which, with specialized hardware and software, has been shown to be capable of reaching the millisecond time scale [163]. Alternatively, enhanced sampling techniques have been developed to facilitate the exploration of proteins’ conformational space [164,165,166], currently inaccessible on standard computational architectures. Along this line, trajectories obtained by very long classical MD or obtained thanks to enhanced sampling methods would provide a set of conformations to be probed by QM/MM simulations.

Moreover, QM/MM simulations may conveniently be used to provide force field parameters for classical MD simulations by means of the force-matching approach [167,168]. This technique, by matching classical forces to those of the QM region of QM/MM trajectories, provides accurate force field parameters, which include environmental and temperature effects for the specific system under study. In this manner, we can envision adaptive simulations, in which cycles of QM/MM (short) and purely MM (extensive) MD simulations alternate [169,170].

*In silico* experiments may include mechanistic studies of enzymatic reactions, and this is certainly a well-established capability of the QM/MM approach. Of increasing interest is also the computation of spectroscopic parameters by means of *ab initio* methods. These latter and, in turn, QM/MM methods are capable of describing any configuration of the system under study with the same accuracy (in contrast with empirical methods, whose accuracy depends on the set of configurations used for the parameterization) and are thus capable of characterizing unusual coordination environments. Spectroscopic parameters computed on those environment may be compared with experimentally measured ones, eventually leading to the assignment to specific molecular structures, from which a hypothesis may be formulated and further experiments designed [12,170,171,172]. Moreover, this computational approach may be used to dissect how protein environmental effects influence and modulate the redox properties of real proteins and bio-inspired compounds [173,174,175].

Finally, the development of more computationally-efficient QM/MM algorithms may allow the use of QM/MM docking approaches, which would overcome the limit of conventional docking methodologies, relying on the charge models of force fields. This is again a major issue when the ligand docking is done at the enzymatic site of metalloproteins, in which the binding site is highly polarized by the metal [176,177,178,179].

In all of these fields, we believe that QM/MM simulations are going to play an ever-increasing important contribution to the investigation of metal-containing proteins and metalloenzymes in the forthcoming years.
